# Seven LncRNA-mRNA based risk score predicts the survival of head and neck squamous cell carcinoma

**DOI:** 10.1038/s41598-017-00252-2

**Published:** 2017-03-22

**Authors:** Zhi-Li Zhang, Li-jing Zhao, Liang Chai, Shui-Hong Zhou, Feng Wang, Yan Wei, Ya-Ping Xu, Peng Zhao

**Affiliations:** 1 Department of otorhinolaryngology,the first affiliated hospital, Zhejiang University School of medicine, 310003 Qingchun Road 79, Hangzhou city, Zhejiang province China; 2 Department of otorhinolaryngology, the second affiliated hospital, Zhejiang University school of medicine, 310003 Qingchun Road 79, Hangzhou city, Zhejiang province China; 3 Department of Oncology, The first affiliated hospital, Zhejiang University School of medicine, 310003 Qingchun Road 79, Hangzhou city, Zhejiang province China

## Abstract

Dysregulation of mRNAs and long non-coding RNAs (lncRNAs) is one of the most important features of carcinogenesis and cancer development. However, studies integrating the expression of mRNAs and lncRNAs to predict the survival of head and neck squamous cell carcinoma (HNSC) are still limited, hitherto. In current work, we identified survival related mRNAs and lncRNAs in three datasets (TCGA dataset, E-TABM-302, GSE41613). By random forest, seven gene signatures (six mRNAs and lncRNA) were further selected to develop the risk score model. The risk score was significantly associated with survival in both training and testing datasets (E-TABM-302, GSE41613, and E-MTAB-1324). Furthermore, correlation analyses showed that the risk score is independent from clinicopathological features. According to Cox multivariable hazard model and nomogram, the risk score contributes the most to survival than the other clinical information, including gender, age, histologic grade, and alcohol taking. The Gene Set Enrichment Analysis (GSEA) indicates that the risk score is associated with cancer related pathways. In summary, the lncRNA-mRNA based risk score model we developed successfully predicts the survival of 755 HNSC samples in five datasets and two platforms. It is independent from clinical information and performs better than clinical information for prognosis.

## Introduction

Head and neck carcinoma (HNSC) is one of the most causes of death in the world^1^, and 600,000 new cases occurred every year, with 50% increasement^2^. Among those patients, more than 90% were identified as squamous cell carcinoma. Smoking, alcohol taking and human papilloma virus (HPV) are all important causes of HNSC carcinogenesis^3^. The prognosis of HNSC currently is unfavorable. The five-year survival rate of HNSC is about 50–60%^4^, and the survival rate of HNSC did not improved in the past 30 years^5^.

Long non-coding RNAs (lncRNAs) are newly discovered non-coding RNAs, with more than 200 nucleotides and no protein coding ability^6^. The gene expression abundance of lncRNAs is much lower than mRNA. Despite of this, lncRNAs play important roles in cell multiple biological processes^7^, including development^8^, diseases^9^, epigenetic modulation^10^, and scaffolding^11^. Recently, studies have emphasized the role of lncRNAs on carcinogenesis and cancer progression^12–16^ among cancer types, including head and neck squamous cell carcinoma^17, 18^. MALAT1 was identified as an oncogene, and the high expression of MALAT1 was associated with metastasis and poor survival across cancers^19–21^. Another lncRNA, DANCR was reported to block the repression effect of miR-214, miR-320a, and miR-199a on CTNNB1 in hepatocellular carcinoma^22^. MEG3 was reported to involved in regulation of epithelial-mesenchymal transition in lung cancer cells^23^. According to recent study on lncRNA GAS5, the allele deletion of rs145204276 was associated with decreased CRC risk and less possibility of lymph-node metastasis^24^. On the other side, mRNAs encode proteins and thus control almost every cell progress. Thus, integrating abundance of lncRNAs and mRNAs enhances the prognostic effect^25^.

In this article, we identified lncRNAs and mRNAs from three independent datasets with Cox univariate regression model, and selected seven lncRNA-mRNAs to develop the risk score with random survival forests variable hunting algorithm. Patients with high risk score have relatively poor prognoses than those with low risk score, in both training and test datasets. The risk score is independent with clinical observations including sex, age, histologic grade, alcohol taking, and primary tumor size. The prognostic performance of the risk score on survival is more favorable than aforementioned clinical information. In summary, the seven lncRNA-mRNA risk score is robust in predicting the survival of HNSC patients, and performs better than clinicopathological information.

## Method and Meterial

### Expression data processing

Processed TCGA expression data was downloaded from TCGA official website (http://cancergenome.nih.gov/), and the upper quantile normalized FPKM (fragments per kilobases per million) values were used. After removing normal samples, genes expressed in over 80% cancer samples were retained. The zero values in the expression matrix were replaced with (minimum non-zero FPKM value)/2 of corresponding gene. Then the expression data were log 2 transformed. The lncRNA expression data was downloaded from TANRIC (http://bioinformatics.mdanderson.org/main/TANRIC:Overview), and processed as mRNAs. GSE41613 data set was downloaded from GEO (https://www.ncbi.nlm.nih.gov/geo/), the raw data were normalized with “cyclicloess”^26^.

Probes were matched to the gene names according to the manufacture provided annotation file. If a single gene matches multiple probes, probes were integrated by using the arithmetic mean to represent the expression level of single lncRNA. The workflow of this work was shown in Figure S1.

### LncRNA and mRNA matching between platforms

Probe target sequences (HG U133 plus 2) was downloaded from Affymetrix website (http://www.affymetrix.com/), and these sequences were mapped to the lncRNA sequences form ENSEMBL (http://ensembl.org/index.html) with bowtie1 software (http://bowtie-bio.sourceforge.net/index.shtml)^27^. This step allows no mismatch and only probes matched to the forward sequences were retained (strand-specific). Probes matched more than one lncRNAs were discarded. For single lncRNAs matching multiple probes, probes were integrated by using the arithmetic mean to represent the expression level of single lncRNA. Totally, 3505 lncRNAs were used for further analysis. Afterwards, the mRNAs and lncRNAs detected in both platforms (NGS and Affymetrix HG U133 plus 2) were retained for further analysis. Z-score was calculated for each gene among samples in each dataset.

### Gene selection, Cox multivariate regression, and resampling

Cox univariate regression^28^ was carried out on TCGA, E-TABM-302, and GSE41613 datasets. LncRNAs and mRNAs significantly associated survival (p < 0.05) in all of these three datasets were retained for further analyses, and 33 mRNAs and lncRNAs identified. Random forest variable hunting algorithm was used for select biomarker combination for prognosis (50 replications and 50 iterations)^29^. Cox multivariate regression model were used to develop the risk score model:

$${\rm{risk}}\,{\rm{score}}=\sum _{i=1}^{n}\beta i\ast xi$$

where β_i_ indicates the coefficient for each gene and x_i_ indicates the z-score transformed relative expression value of each gene.

Resampling was carried out, 80% of all samples randomly selected. After dividing these samples into high/low risk group, survival difference (p values) was calculated. Repeat this step for 10,000 times, distribution of p values (survival of high-risk vs low-risk) was plotted (Figure S2).

### Statistical analysis

All statistical analysis were performed with R (https://www.r-project.org/, v3.0.1) and R packages. Normalization of Affymetrix microarray data were implemented with R package “limma”^26^. The survival analysis, cox univariate regression, cox multivariate regression and cox probability hazard model were carried out on R package “survival”^30^. Random forest variable hunting was performed with package “randomForestSRC”. The ROC curve was plotted with R package “pROC”^31^, and the nomogram were plotted with R package “rms”^32^. The Gene Set Enrichment Analysis was implemented with java software GSEA (http://software.broadinstitute.org/gsea/index.jsp)^33^.

## Results

### Identification of survival related mRNAs and lncRNAs

**Figure 1:**
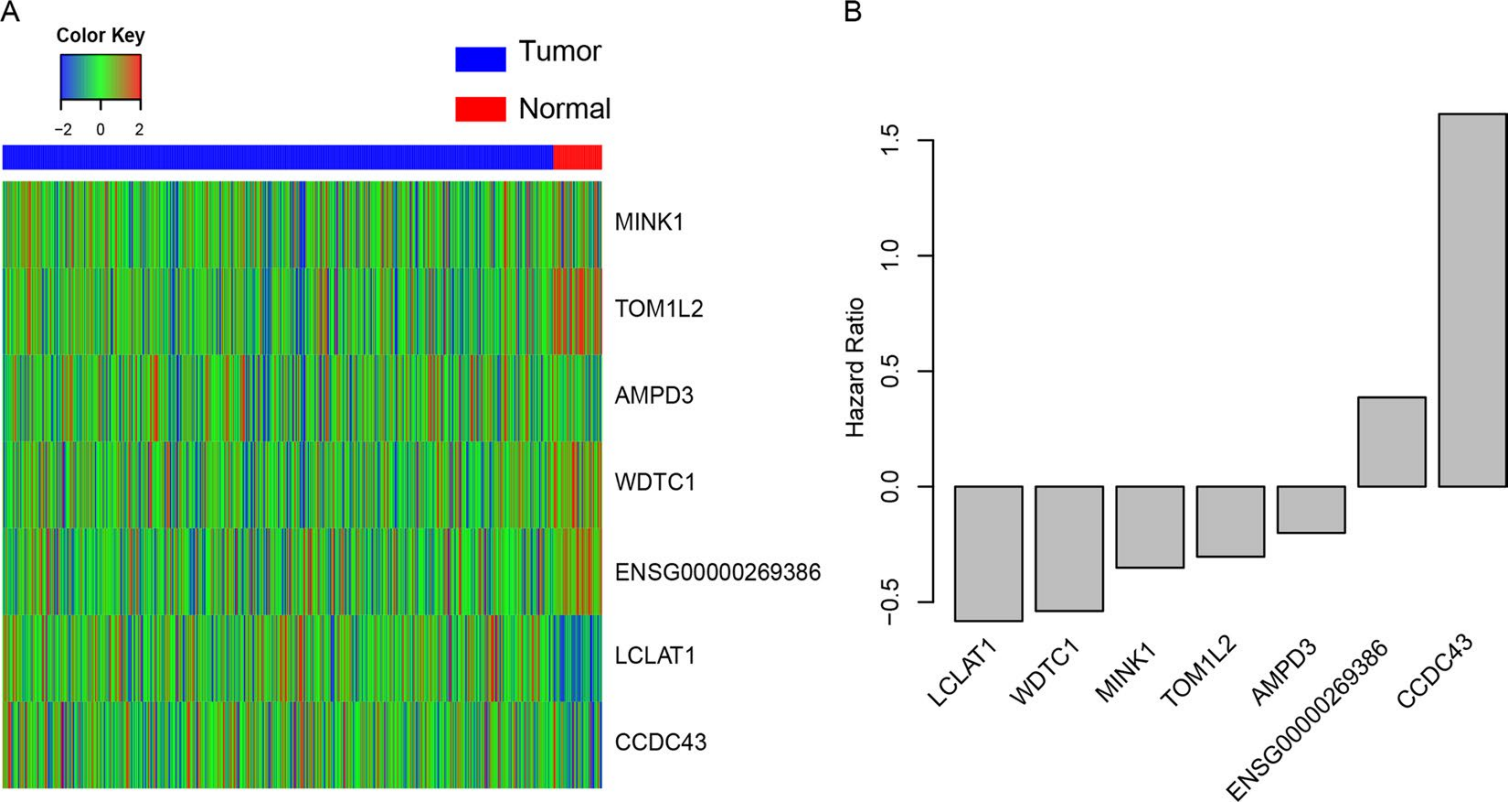
Seven mRNAs and lncRNA identified. (**A**) The expression pattern of the normal tissues (red) and tumor tissues (blue) is different. (**B**) The hazard ratio of seven RNAs.

In order to avoid result-data overfit, cox univariate regression was performed in three independent datasets, including TCGA (N = 407, overall survival), GSE41613 (N = 97, overall survival), and E-TABM-302 (N = 81, overall survival). Significant survival related mRNAs and lncRNAs (p < 0.05) in all of these datasets were identified, which yields 33 genes, including 2 lncRNAs and 31 mRNAs. To narrow down the panel, random forest variable hunting algorithm was implemented and 7 genes, including 6 mRNAs (LCLAT1, WDTC1, MINK1, TOM1L2, AMPD3, CCDC43) and 1 lncRNA (ENSG00000269386), were selected. These genes are differentially expressed between normal and tumor tissues (Fig. 1A). The hazard ratio of each gene was evaluated (Fig. 1B). The hazard ratios of LCLAT1 and CCDC43 are positive, indicating the anti- survival function of these genes, while the coefficients of other 5 genes are negative, including ENSG00000269386 (RAB11B-AS1), TOM1L2, AMPD3, MINK1 and WDTC1, indicating that they may be tumor suppressor genes.

### Performance of risk score in training dataset

**Figure 2:**
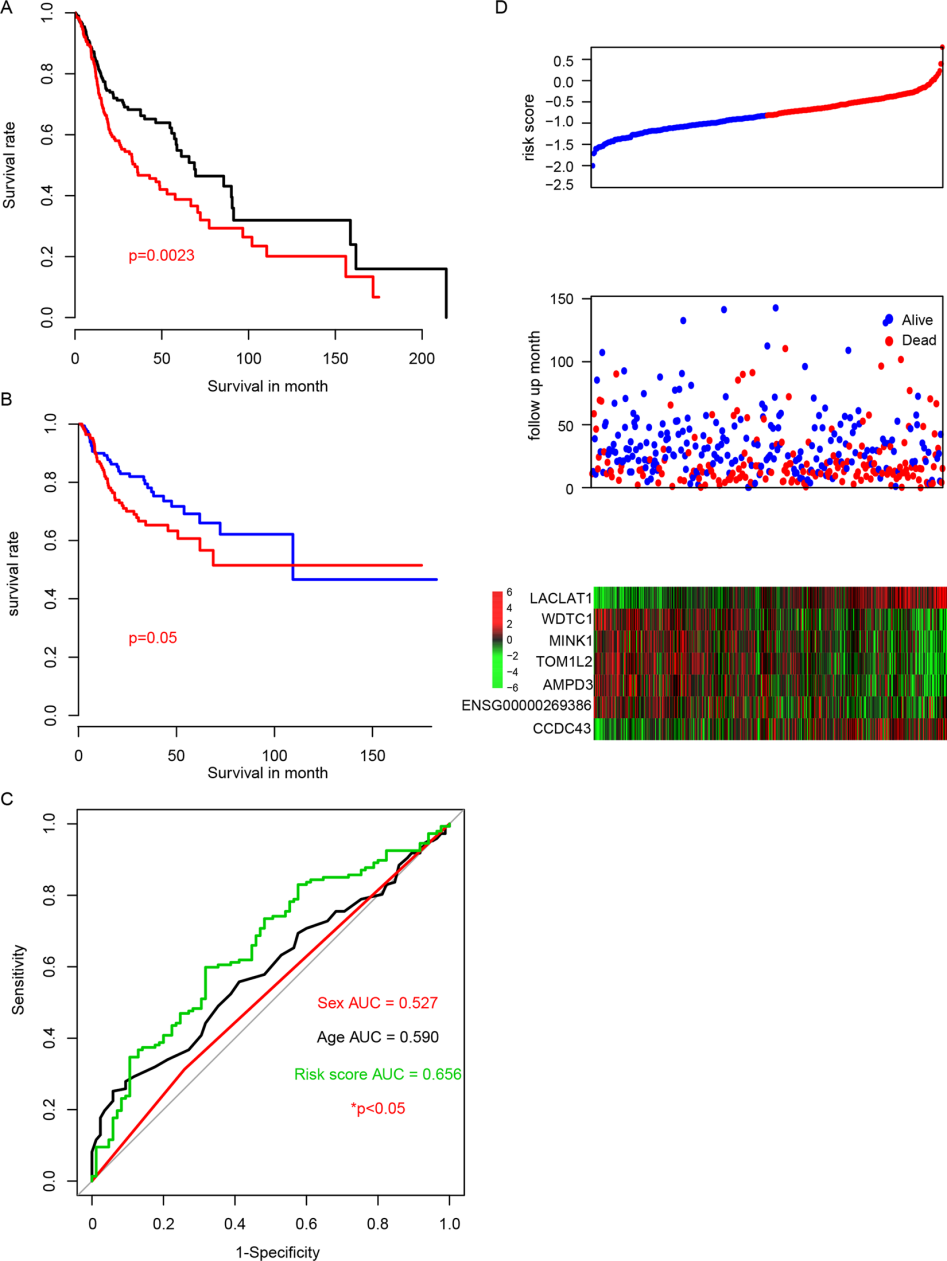
The risk score performance in the TCGA (training) datasets. The overall survival of high risk score group and low risk score group (**A**), and the recurrence-free survival between high/low risk score groups (**B**). The 3-year survival receiving operating characteristic curve (ROC) of age (black), gender (red) and risk score (green) (**C**). (**D**) The relationship between risk score (upper), survival information (middle) and z-score transformed expression value (bottom) was shown.

To integrate all these seven genes selected in our previous step, a cox multivariable regression model was developed on the largest cohort, TCGA dataset. The risk score of each patient were identified as the following: risk score = (1.6136310285262 × LCLAT1) + (−0.582470134280548 × WDTC1) + (−0.351319390355873 × MINK1) + (−0.538752453473946 × TOM1L2) + (−0.303600743722099 × AMPD3) + (−0.200539523626066 × ENSG00000269386) + (0.386646135470189 × CCDC43). The corresponding p-values were shown in Table 1. The risk score for each patient in the TCGA dataset was calculated, and high risk group/low risk group were divided using the median risk score as cutoff. We evaluated the survival of the high risk group and low risk group, and the survival time of high risk group is significantly shorter than the low risk group (Fig. 2A). In addition, the tumor-free survival (TFS) difference between high/low risk group was evaluated, and the result shows high risk group has a significantly less tumor-free survival rate, in consistent with the overall survival (Fig. 2B). To avoid overfit due to cancer heterogeneity^34^, resampling (80% samples, 10,000 repeats) was used to evaluate the performance of risks score, and the results indicates that 97.8% resampling was significantly associated with risk score (p < 0.05), as shown in Figure S1. And according to the three-year survival receiving operating characteristic (ROC) curve, the area under curve (AUC) of risk score reached 0.66 (Fig. 2C), which is significantly higher than other clinical information, indicating that the seven lncRNA-mRNA based risk score is a good indicator for prognosis. The detailed risk score, seven lncRNA-mRNA expression and survival information was shown in Fig. 2D.

**Table 1:** The Coefficients and p values of each gene.

Gene	Coefficients	p value
LCLAT1	1.613631029	0.024
WDTC1	−0.582470134	0.036
MINK1	−0.35131939	0.05
TOM1L2	−0.538752453	0.006
AMPD3	−0.303600744	0.019
ENSG00000269386	−0.200539524	0.018
CCDC43	0.386646135	0.0054

P values were generated from univariate cox regression in TCGA dataset.

### Performance of risk score in test datasets

**Figure 3:**
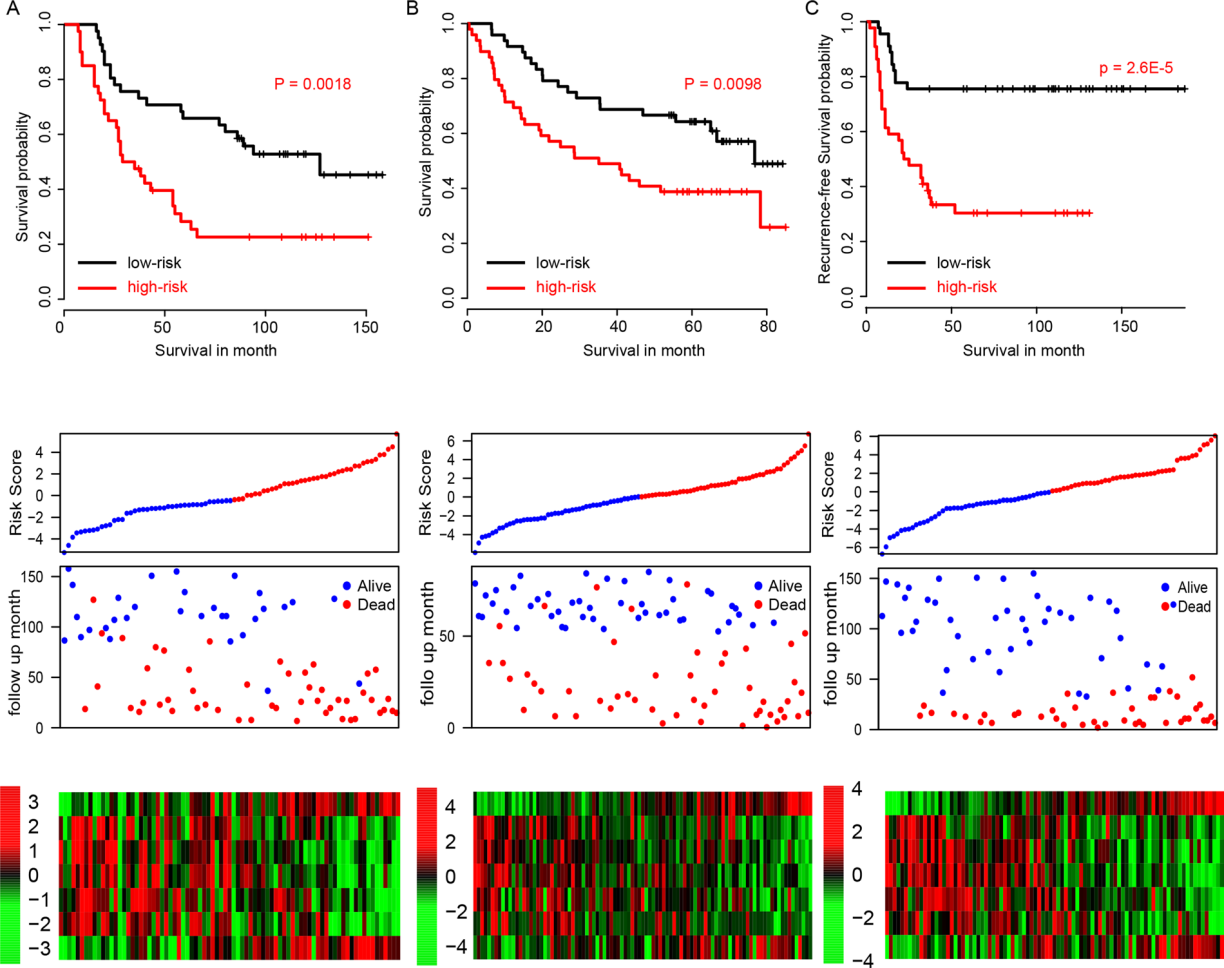
The survival information of three independent test datasets. The survival plot (upper panel), risk score (2^nd^ panel), survival information (3^rd^ panel) and z-score transformed expression value (top-down, LACLAT1, WDTC1, MINK1, TOM1L2, AMPD3, ENSG00000269386, CCDC43) were shown in E-TABM-302 (**A**), GSE41613 (**B**), and E-MTAB-1328 (**C**).

To further validate the robustness of lncRNA-mRNA risk score model developed in the TCGA dataset (RNA-seq), the performance of the risk score was also evaluated in three independent datasets (microarray), E-TABM-302 (N = 81) and GSE41613 (N = 97) using coefficients in training datasets. The high risk and low risk groups were also divided according to the median risk score. The overall survival time in high risk group was significantly shorter than that in low risk group, in both datasets (Fig. 3A,B). Considering that the expression of candidates lncRNAs and mRNAs were generated from those two datasets, over fit may exists. We utilized another totally independent datasets, E-MTAB-1328 (N = 89) for further validation. The tumor-free survival profile and gene expression pattern resemble our previously conclusion (Fig. 3C).

We also randomly selected seven genes from 33 previously identified genes, and developed a risk score model with these genes. However, these models are not robust in the other validation datasets (Table S1), indicating that our method performs better. In summary, our results indicate that the seven lncRNA-mRNA based risk score is robust across platforms and datasets.

### Risk score and clinicopathological information for prognosis

**Figure 4:**
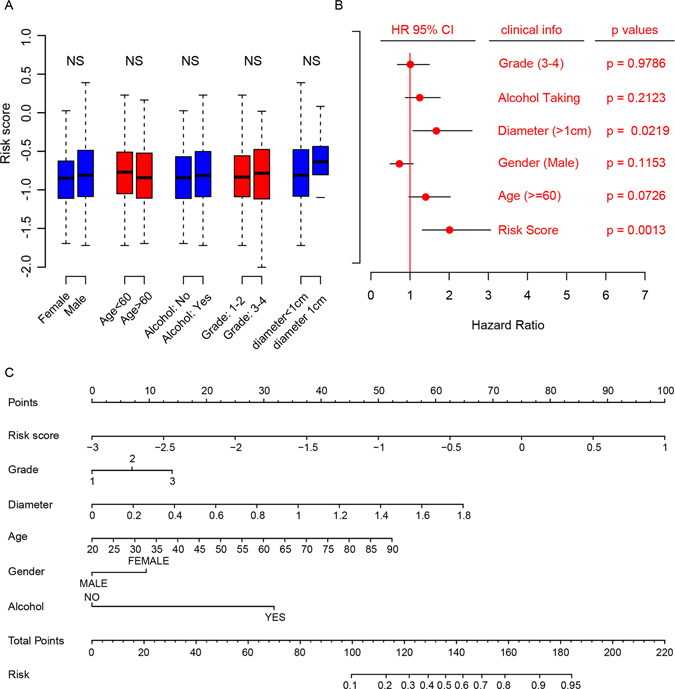
The relationship between risk score and other clinical information. (**A**) The correlation between risk score and clinical information is not significantly different (p > 0.05, NS). (**B**) Cox multivariate regression with clinical information and risk score for survival. (**C**) Nomogram for predicting the 3-year event (death) with risk score and clinical information.

Correlation analyses were implemented between risk score and clinical indicator, which include gender, age, alcohol taking, histologic grade, diameter of primary tumor, on the largest cohort, TCGA dataset. As show in Fig. 4A, none of these clinical information is associated with our seven lncRNA-mRNA based risk score, indicating that the risk score is independent from the clinical information.

Furthermore, to identify the importance of the clinicopathological information and the risk score, cox multivariable probability hazard model was employed (Fig. 4B). The risk score is the most significant correlated with the survival information (p = 0.0013) and highest median risk score (HR = 2, 95% CI = 1.2–3.1), indicating that the risk score performs better than the other clinical information. To facilitate the utilization of risk score, a 3-year survival nomogram were plotted (Fig. 4C) considering risk score and aforementioned clinicopathological observations. In consistent with our cox multivariable regression results, the risk score contributes the most risk points (ranged 0–100), whereas the other clinical information contributes much less (diameter of primary tumor, ranged 0–65).

Altogether, these results indicate that the seven lncRNA-mRNA based risk score is independent from the clinical information, and performs better in the survival prediction than that clinical information.

### Risk score and radiation therapy

**Figure 5:**
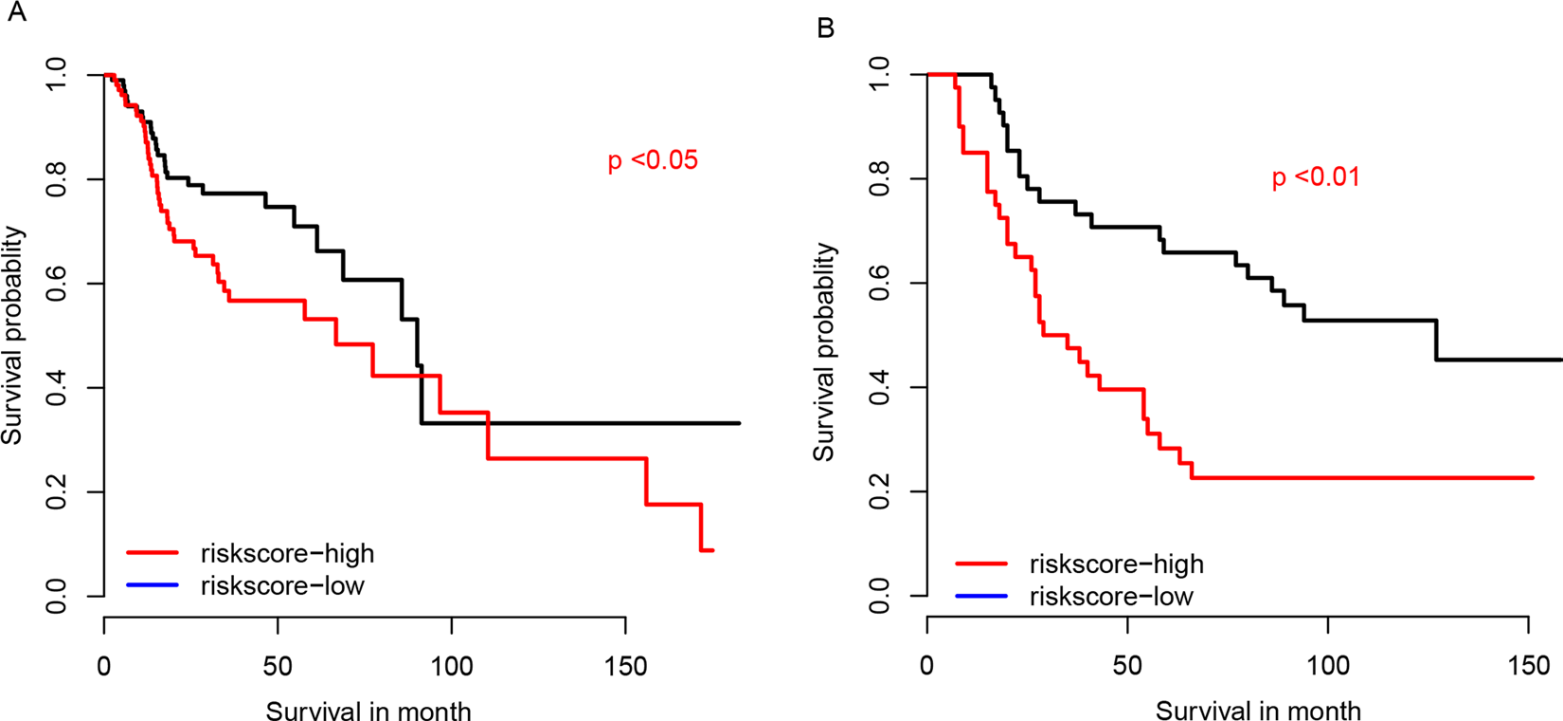
The survival rate of patients with radiation therapy. The survival of patients received radiation in TCGA (**A**) and E-TABM-302 datasets (**B**).

Radiation therapy is the most important adjuvant HNSC treatment method. Thus, the correlation between risk score and the radiation outcome were evaluated. We used two independent datasets providing the radiation therapy information to test whether our risk score is also available for survival prediction of patients underwent radiation therapy. As we expected, the patients with high risk score who received radiation had a worse prognosis in both TCGA (Fig. 5A) and E-TABM-302 (Fig. 5B) dataset, compared to the low risk group. These results indicate that the risk score is also available for the prognosis of HNSC patients with radiation therapy.

### Altered pathways in high and low risk score group

**Figure 6:**
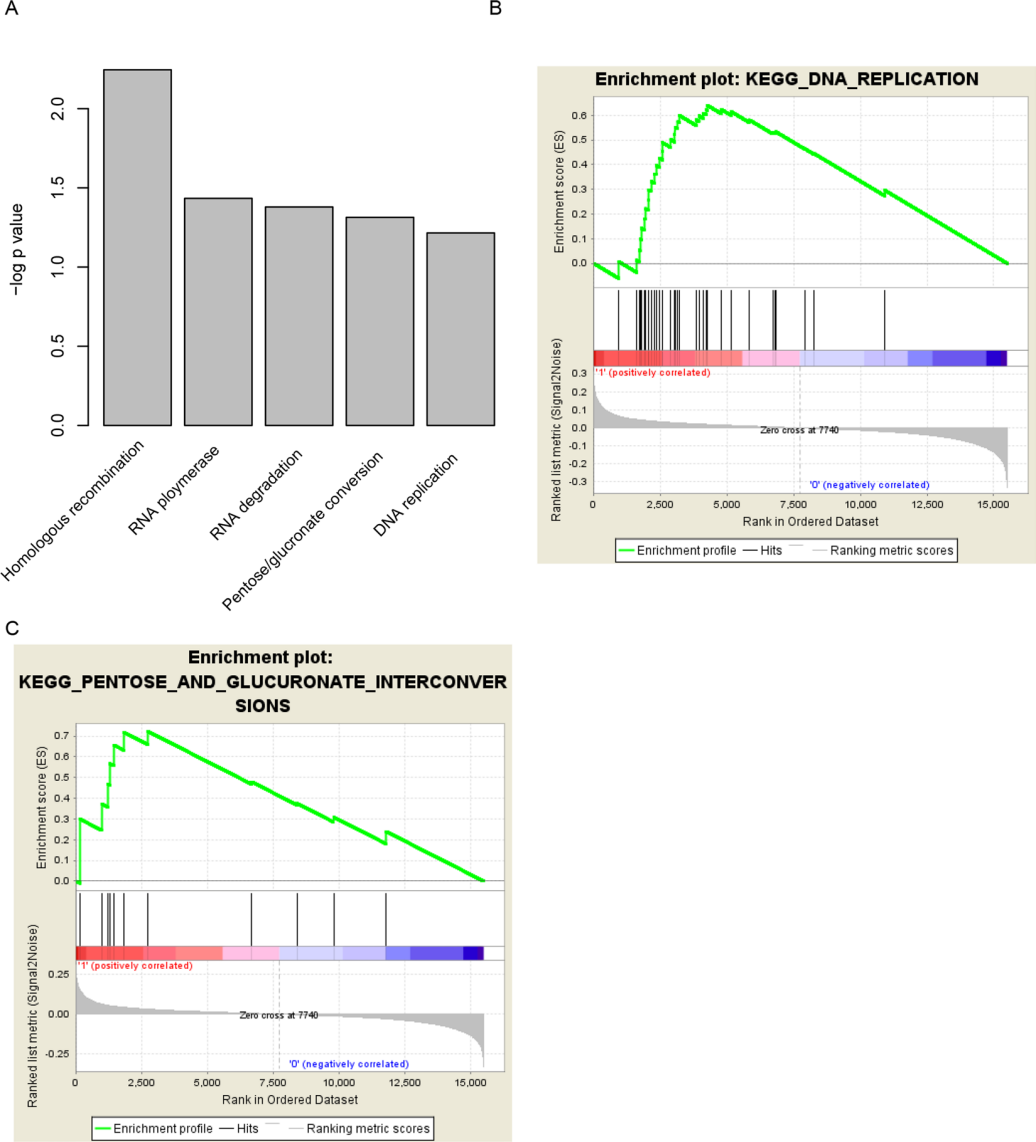
KEGG pathways associated with risk score. The pathways associated with risk score (**A**), include DNA replication (**B**) and pentose/glucoronate conversion (**C**).

To investigate the potential altered pathways in the high risk group, Gene Set Enrichment Analysis (GSEA) was implemented between high/low risk groups. According to the results, we noticed that KEGG pathways including “homologous recombination”, “RNA polymerase”, “RNA degradation”, “pentose and glucoronate interconversions” and “DNA replication” were significantly enriched (p value < 0.01, Fig. 6A). Of these pathways, DNA replication and pentose pathways were noted for its role in carcinogenesis and cancer maintenance (Fig. 6B,C), suggesting that the seven lncRNA-mRNA based risk score may reflect the status of these cell processes, and thus predict the survival of HNSC patients.

## Discussion

The heterogeneity of head and neck squamous carcinoma is high according to a lot of studies^35, 36^, which may due to the heterogeneity of causes of HNSC^37^, including alcohol abusing, HPV infection and diet. It makes the prognosis and therapy of head and neck squamous cell carcinoma difficult. In the past, the prognosis of HNSC is largely depend on the TNM staging system and histologic grade^38^. However, the effect is unfavorable.

The clinical prognostic effect of mRNAs in HNSC have been widely reported in the past decades. Recently, the utilization of lncRNAs as prognostic marker and potential therapy targets has been reported^39^, including head and neck squamous cell carcinoma^40, 41^. The usage of lncRNAs for disease association have been reported in the past years^7, 9, 37, 39^, and achievements have been obtained. However, the combination of lncRNAs and mRNAs is currently not reported for prognosis, to our knowledge. In current work, we developed a cox multivariate regression model with survival-related genes and random forest variable selection. Afterwards, the performance of the seven lncRNA-mRNA based risk score were assessed in the training dataset and validated in another two datasets, and further validated in a totally independent dataset. Patients in the high risk group significantly have a shorter survival than the low risk group. And more, the seven lncRNA-mRNA based risk score is independent from the clinicopathological observations and performs better than this information.

The pathways significantly associated with the risk score were “RNA polymerase” and “DNA replication”, suggesting that our risk score may reflects the basic status of HNSC cells, including DNA replication and basic metabolism.

## Electronic supplementary material

Supplementary information
