# Supplementary material for: Seven LncRNA-mRNA based risk score predicts the survival of head and neck squamous cell carcinoma
Source: Sci Rep. 2017 Mar 22;7:309. doi: 10.1038/s41598-017-00252-2 (PMC5428014; doi:10.1038/s41598-017-00252-2)
Supplement: Supplementary file 1 — Supplementary information [file 41598_2017_252_MOESM1_ESM.pdf]

Seven LncRNA-mRNA based risk score predicts the survival of head and neck squamous cell carcinoma

Zhi-Li Zhang[1,*], Li-jing Zhao[2], Liang Chai[1], Shui-Hong Zhou[1], Feng Wang[1], Yan Wei[1], Ya-Ping Xu[1], Peng Zhao[3]

[1]ENT Department, the first affiliated hospital of medical college, Zhejiang University. 310003, Qingchun Road 79, Hangzhou city, Zhejiang province, China.

[2]ENT Department, the second affiliated hospital of medical college, Zhejiang University. 310003, Qingchun Road 79, Hangzhou city, Zhejiang province, China

[3]Oncology Department, The first affiliated hospital of medical college, Zhejiang University. 310003, Qingchun Road 79, Hangzhou city, Zhejiang province, China

[*]Corresponding author: zhangzhili_zju@126.com

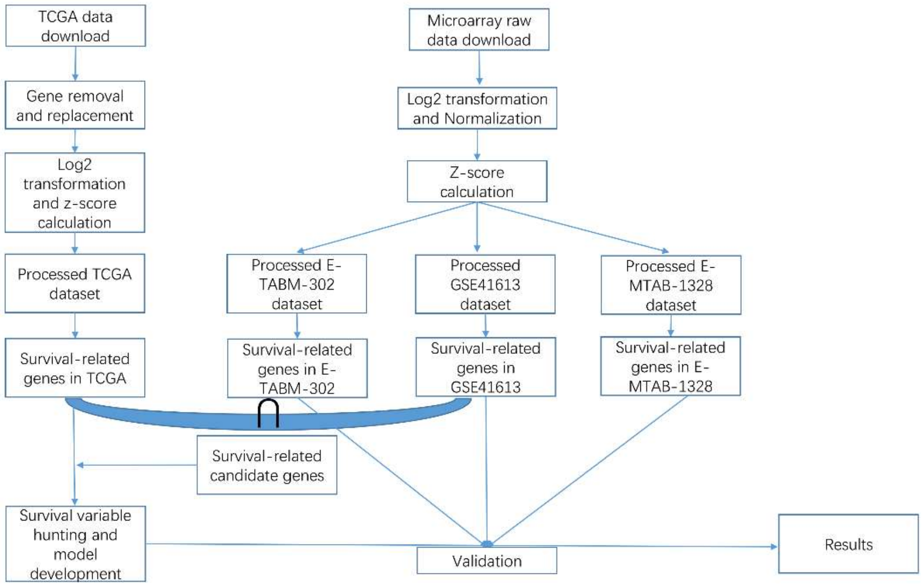

Figure S1. The workflow of this article.

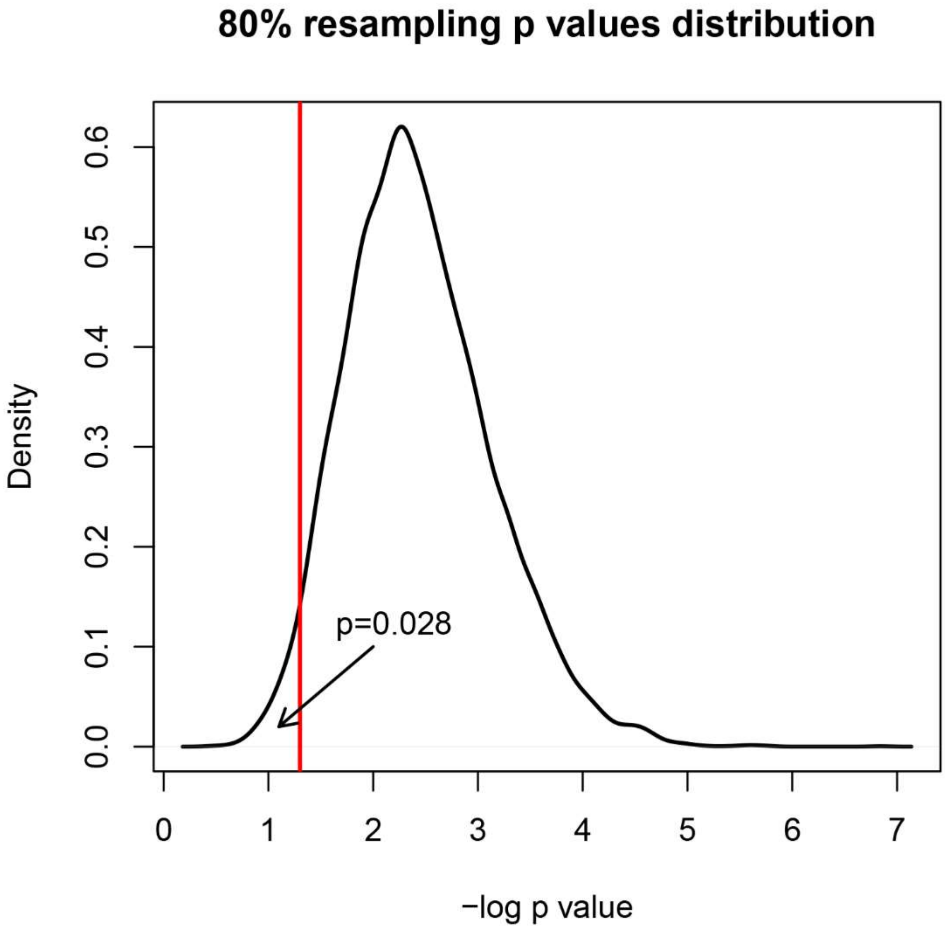

Figure S2. The distribution of p values of 10000 times 80% resampling. The x axis indicates the log 10 transformed p values, and y axis indicates the density of p value distribution. The red line is $\log_{10}0.05$.

Rank	TCGA (train)	E-MTAB302	GSE41613	E-TABM-1328	number_significant
1	2.40E-07	0.52	0.037	0.031	3
2	0.01	0.37	0.032	0.22	1
3	2.50E-05	0.18	0.33	0.15	1
4	0.00077	0.12	0.00063	0.22	2
5	1.40E-02	0.18	0.004	0.46	2
6	1.20E-06	0.0013	0.16	0.19	2
7	2.30E-03	0.08	0.07	0.88	3
8	9.80E-06	0.12	0.016	0.053	3
9	3.40E-03	0.65	0.044	0.16	2
10	5.60E-04	0.08	0.0097	0.031	3

Table S1. The p values of ten times of randomly selected 7-gene combinations.
